# Atomistic Investigation on the Wetting Behavior and Interfacial Joining of Polymer-Metal Interface

**DOI:** 10.3390/polym12081696

**Published:** 2020-07-29

**Authors:** Mingyong Zhou, Liang Fu, Fengze Jiang, Bingyan Jiang, Dietmar Drummer

**Affiliations:** 1 College of Mechanical and Electrical Engineering, Central South University, Changsha 410083, China; fuliangcsu@csu.edu.cn; 2 Institute of Polymer Technology (LKT), Friedrich-Alexander-University Erlangen-Nürnberg, Am Weichselgarten 9, 91058 Erlangen-Tennenlohe, Germany; fengze.jiang@fau.de (F.J.); dietmar.drummer@fau.de (D.D.); 3 State Key Laboratory of High Performance Complex Manufacturing, Central South University, Changsha 410083, China

**Keywords:** polymer-metal hybrid, molecular dynamics simulation, wetting behavior, interfacial interaction, injection-molded direct joining

## Abstract

Polymer-metal hybrid structures can reduce the weight of components while ensuring the structural strength, which in turn save cost and subsequently fuel consumption. The interface strength of polymer-metal hybrid structure is mainly determined by the synergistic effects of interfacial interaction and mechanical interlocking. In this study, the wetting behavior of polypropylene (PP) melt on metal surface was studied by molecular dynamics simulation. Atomistic models with smooth surface and nano-column arrays on Al substrate were constructed. Influences of melt temperature, surface roughness and metal material on the wetting behavior and interfacial joining were analyzed. Afterwards the separation process of injection-molded PP-metal hybrid structure was simulated to analyze joining strength. Results show that the initially sphere-like PP model gradually collapses in the wetting simulation. With a higher temperature, it is easier for molecule chains to spread along the surface. For substrate with rough surface, high density is observed at the bottom or on the upper surface of the column. The contact state is transitioning from Wenzel state to Cassie–Baxter state with the decrease of void fraction. The inner force of injection-molded PP-Fe hybrid structure during the separation process is obviously higher, demonstrating a greater joining strength.

## 1. Introduction

Lightweight metallic materials, such as aluminum (Al), magnesium (Mg) and titanium (Ti) are widely used in the applications of automobile, aerospace and electronics because to their high specific stiffness, weight reduction and wear resistance. However, due to the inherent characteristics in some cases, these materials have shortcomings in thermal conductivity, processing complexity and high cost of raw material. On the other hand, polymer and plastic material are playing dominant roles in the modern industrial economy and the social life, mainly with the benefits of simple molding, low cost and light weight. Nevertheless, polymer and plastic products have the disadvantages of low mechanical strength, poor dimensional and thermal stability. By combing the advantages of metal and polymer materials, the polymer-metal hybrid structure can greatly reduce the weight of components while ensuring the structural strength, which in turn saves cost and subsequently fuel consumption [1,2,3,4,5,6].

Injection molding, or the direct joining method is one of the most commonly used technologies in the fabrication of polymer products [7,8,9]. With metallic part as an insert assembled in the cavity of injection mold, the polymer-metal hybrid structure can be easily fabricated in mass production. In order to enhance the bonding strength at the interface, roughness treatments such as chemical etching, laser ablation and plasma treatment are usually performed on the surface of metal insert prior to the injection molding process [8,10,11]. Among these applications, metals including Al, Mg, nickel (Ni), iron (Fe) and their alloys are the most commonly used materials for polymer-metal hybrid structure [12,13,14]. In terms of polymer material, polypropylene (PP) is one of the typical polymer materials. It is also widely used as the matrix in composite material system and in the application of automobile industry. Li et al. [15] proposed an injection method based on metal surface topography for producing A6061 alloy and PP hybrid structures. In this research, the contact angles of polymer melt on different surface topographies and at different metal temperatures were introduced to analyze the bonding mechanism experimentally.

In direct joining process, there is no chemical treatment in order to form chemical bonding. Therefore, the joining strength is mainly determined by the synergistic effects of mechanical interlocking and non-bonded interaction at the interface. However, conventional interface analysis based on macroscopic scale needs data about the mechanical behavior of the interphase that is difficult to obtain from direct measurement. Recently, molecular dynamics (MD) simulation has attracted great attention in the field of interfacial interaction and molding process at nanoscale [16,17,18,19]. It has become a promising tool for observing the atomic movements that are allowed to interact and giving a view of the dynamic evolution at atomic level. Moyassari et al. [20,21] used coarse-grained MD simulations method for the short-chain branched bimodal polyethylene and the linear polyethylene blend systems to investigate the topological features and mechanical behaviors of the semi-crystalline polymer. By using the fully atomistic MD simulations with the reactive force field (ReaxFF), Rout et al. [22] studied the interface joining of metal and polymer during the mechanical friction process in order to check whether chemical bond between polymer and metal is formed. In terms of the wetting behavior simulation, most attention has focused on water droplet or metal in liquid state on different substrates by MD method [23,24,25]. Few literatures are reported on the contact angle of polymer droplet on metallic or soft substrate [26,27,28]. The spreading process of polymer droplet and the interaction at the polymer-metal interface still need more investigations.

In our previous research, the mechanical interlocking at the polymer-metal interface was investigated [29]. Therefore, this paper aims to explore the wetting behavior of polymer melt on metal surface and then to investigate the joining strength at the interface. Simulation models with smooth surface and nano-column arrays on Al substrate were constructed, respectively. The spreading process, height of mass center, droplet base radius, contact angle and interaction energy were introduced to investigate the wetting property. Influences of melt temperature, surface roughness and metal material on the wetting behavior and interfacial joining were analyzed. After that, the separation process for the injection-molded PP-metal hybrid structure was then simulated to analyze the joining strength induced by different substrate materials. The interaction energy at the interface, the radius of gyration and the inner force in PP layer were utilized to explain the interfacial joining. The output of this study will provide useful reference for material selection and future design in the polymer-metal hybrid structure.

## 2. Materials and Methods

### 2.1. Materials and Model Constructing

**Figure 1:**
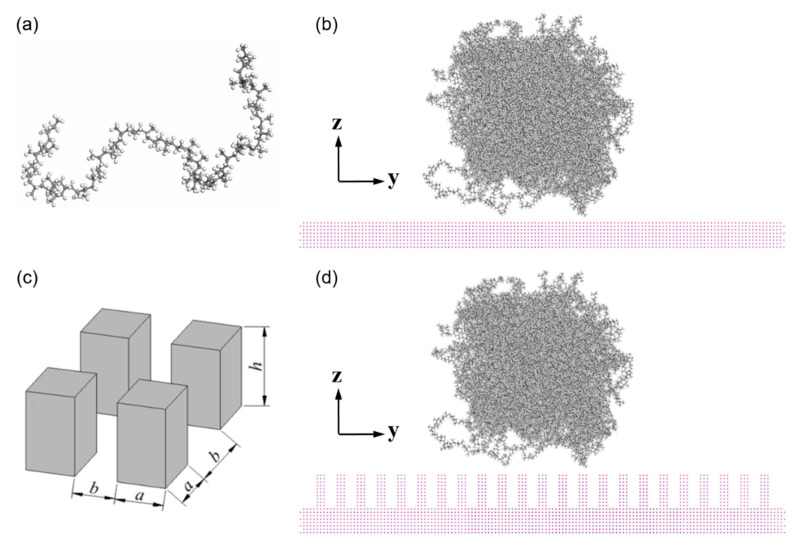
Atomistic models for wetting behavior of PP droplet on the metallic substrate. (**a**) a single chain with the degree of polymerization of 50, (**b**) PP model on the metal substrate with smooth surface, (**c**) nano-column arrays on metal surface and (**d**) PP model on the metal substrate with nano-column arrays.

Before the construction of wetting behavior system, polymer and metal layers were separately prepared. It is known that the content of isotactic structure is about 95% in PP resin produced in general industry. Therefore, in this atomic study, isotactic PP model was constructed in a cube with the dimensions of 8.0 nm × 8.0 nm × 8.0 nm. It is reported that PP has a semicrystalline structure with different crystallite sizes (>10 nm) and the amorphous phase [20,21]. In this present work, the PP structure was simplified as amorphous phases in small calculated volumes. The degree of polymerization is suggested to be a relatively low value, otherwise the PP model is difficult to form an ideal spherical droplet for the contact angle analysis in the following simulation. Therefore, the degree of polymerization was set to be 50, with an initial density of 0.85 g/cm^3^ in PP model, as shown in Table 1 and Figure 1a,b. There are in total of 120 chains and correspondingly 54240 atoms in this model. Geometry optimization and subsequently five-circle anneal treatments from 373 K to 573 K for PP model were carried out to optimize the conformation of molecule structure. The PP model was then heated up to the melt temperature of 503 K to access a stable state.

As mentioned in the introduction section, aluminum (Al), nickel (Ni) and iron (Fe) were selected as the metallic substrates in this present study, which was named as P0. Three different substrates were built, respectively, with the same dimensions of 20.0 nm × 20.0 nm × 1.4 nm in length, width and height. The metal substrates were kept at a same temperature with the PP model. The interface system for MD simulation was then constructed by assembling the PP model and metallic substrate layer together. In order to further investigate the influence of surface roughness on the wetting behavior, nano-column arrays with different dimensions were built. These nanostructured surfaces were named as P1, P2, P3 and P4, based on the dimensional parameters of the column arrays, as shown in Table 2. The height (h) of column was 2.02 nm and the distance between two neighboring columns (a + b) was 1.72 nm, while the side length (a) ranges from 0.40 nm to 1.21 nm, as shown in Figure 1c. All the metal atoms were kept constrained so that the metal layer was treated as a rigid body since the metal substrate has a much higher stiffness than those of polymer.

### 2.2. Force Field and Simulation Procedure

In this simulation, polymer consistent force field (PCFF) was adopted to describe the intermolecular and non-bonded interactions between atoms in polymer layer. The force field consists of not only the bond stretching, angular bending and torsion potential, but also non-bonded interactions such as the 9-6 Lennard–Jones and Coulomb potential. The non-bonded interaction between the PP model and metallic substrate layer was also described by the Lennard–Jones potential and the Lorentz–Berthelot combining rules, with the cut-off distance for the non-bonded interaction of 1.25 nm.

Prior to the wetting behavior simulation, both PP model and metal substrate were pre-heated at a varied temperature, that is 503 K, 473 K, 443 K, 413 K and 383 K. The simulation process was then undertaken within a total of 6.5 ns in a constant particle number, volume and temperature (NVT) ensemble, first 500 ps with a time step of 1 fs and then 6.0 ns with a time step of 1.5 fs. Non-periodic boundary conditions were utilized during the simulation, so that the interaction between atom from the top of PP model and atom at the bottom of substrate layer can be efficiently avoided. The contact angle of polymer droplet on the metal surface was measured by geometric method [23,30], drawing a circle around the droplet and then a tangent line to the solid-liquid interface by using the AutoCAD software (Autodesk Inc., San Francisco, CA, USA). The contact angle of each image is analyzed four times to obtain the mean value and standard deviation of the data. After that, the separation process for the injection-molded PP-metal hybrid structure was then simulated to further investigate the interfacial joining. All the simulations mentioned above were performed by the large-scale atomic/molecular massively parallel simulator (LAMMPS, Sandia National Laboratories, Livermore, CA, USA) [31], an open source molecular dynamics package in a computer cluster with Intel Xeon Scalable Silver (Intel Corp., Santa Clara, CA, USA) 4216 processor and 64 cores parallelization. The open visualization tool OVITO (OVITO GmbH, Darmstadt, Hessen, Germany) program was used for the visualization and analysis.

## 3. Results and Discussion

### 3.1. Wetting Behavior of PP Droplet on Smooth Surface

**Figure 2:**
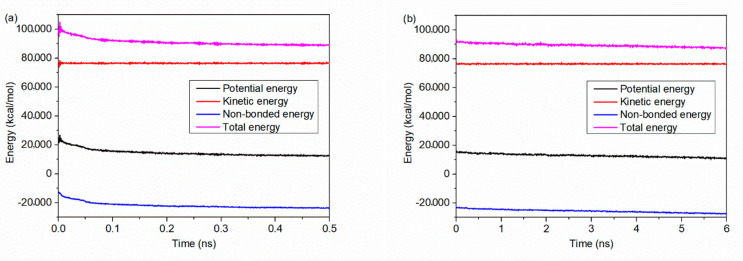
Energy distribution as a function of simulation time during (**a**) the first 0.5 ns and (**b**) the later 6.0 ns. The potential energy, kinetic energy, non-bonded energy and the total energy are calculated, respectively.

The potential energy, kinetic energy, non-bonded energy and the total energy are calculated, as a function of simulation time. As mentioned above, the wetting simulation is undertaken in the NVT ensemble, which means the whole system is kept under a controlled temperature. Since the kinetic energy is related to the temperature, little change in kinetic energy is observed, as shown in Figure 2. Results from the initial 0.5 ns simulation shows that the potential energy, non-bonded energy and the total energy first drop rapidly at the beginning. After that, the trend of change is slowed down. As seen in Figure 2b, the change in each energy become much weaker and gradually trend to a steady state, which demonstrate that a thermodynamic equilibrium is basically reached within a total simulation time of 6.5 ns.

**Figure 3:**
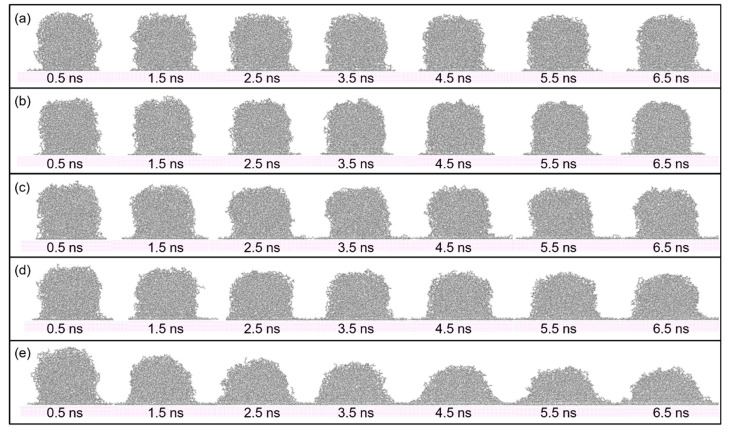
Snapshots of the spreading process of PP droplet with simulation time on the smooth surface. The temperature of PP droplet was (**a**) 383 K, (**b**) 413 K, (**c**) 443 K, (**d**) 473 K and (**e**) 503 K respectively.

Figure 3 shows seven sequential snapshots of the spreading process with simulation time. The change in melt temperature of PP droplet, ranging from 383 K to 503 K, result in different spreading speeds on the Al surface. By comparing the spreading process at different temperatures, it is found that molecule chains in PP model are easier to spread along the surface with a higher temperature. This is because the rising of temperature can increase the kinetic energy in PP model. At the beginning of the spreading process, the PP droplet forms a sphere-like structure while atoms in the bottom of PP model start to spread out along the Al surface. As the time goes on, the whole PP model gradually collapses and more atoms are attached to the Al surface, mainly because of the molecular diffusion and interaction force with the substrate.

**Figure 4:**
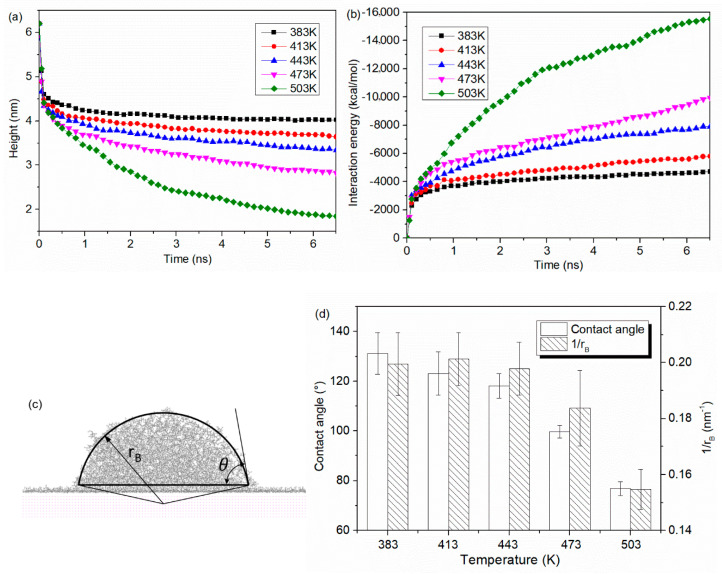
(**a**) height of mass center and (**b**) interaction energy as a function of simulation time, (**c**) calculation of contact angle and base radius and (**d**) contact angle and base radius of PP droplets on Al surface at different temperatures. The temperature of PP droplet was ranging from 383 K, 413 K, 443 K, 473 K to 503 K.

For PP droplet, the height of mass center is calculated during the spreading process. It is shown in Figure 4a that the height of PP droplet is gradually decreasing with the simulation time. With a higher temperature, the height deceases more rapidly. For instant, when the temperature is 383 K, the height of PP droplet is about 4.02 nm at 6.5 ns, while the height of mass center decreases to 1.83 nm when the temperature is 503 K. The droplet becomes flat and hydrophilic to the Al surface with the rising temperature. The contact angle and base radius of droplet were also calculated, as shown in Figure 4c. Similar results can be also observed in Figure 4b,d. The decrease in the height of mass center means an increase in effective contact at the interface, which reflects an increase in droplet base radius and, correspondingly, an increase in the spreading ability of PP droplet. As a result, when the melt temperature is 503 K, the base radius reaches to a maximum value of 6.47 ± 0.30 nm. According to the modified Young’s equation [32], the relationship of contact angle (*θ*) and droplet base radius (*r_B_*) can be described in Equation (1):

(1) $$cos\;\theta =cos\;\theta_{\infty }-\frac{\tau }{\gamma_{LV}}\times \frac{1}{r_{B}}$$

where $\gamma_{LV}$ is the surface tension, $\theta_{\infty }$ is the contact angle corresponding to an infinitely large droplet and τ is the free-energy contribution. As demonstrated in Equation (1), the contact angle is linearly related to the $1/r_{B}$. It can be shown in Figure 4c that the contact angle of PP droplet at 6.5 ns is about 131.2 ± 8.3° (*T* = 383 K), 123.0 ± 8.7° (*T* = 413 K), 118.2 ± 4.9° (*T* = 443 K), 103.6 ± 6.0° (*T* = 473 K), 76.8 ± 2.8° (*T* = 503 K). With the rising temperature, the wetting property of PP droplet first shows hydrophobicity and then turn to be hydrophilic characteristics. Consistent regulations of the influence of melt temperature on the contact angle can be also found in the experimental researches [15,33,34]. PP droplet with high melt temperature can decrease the melt viscosity and flow resistance, which favors the wetting property and improves the interfacial joining between two phases. When the molecule is spreading on the Al surface, non-bonded interactions between PP and Al atoms are gradually generated. The interaction energy is defined by the Equation (2):

(2) $$E_{inter}=E_{total}-(E_{polymer}+E_{metal})$$

where $E_{total}$ is the total potential energy of the polymer-metal system, $E_{polymer}$ is the potential energy of polymer without Al layer and $E_{metal}$ is the potential energy of Al layer without the polymer. Figure 4b illustrates the interaction energy at the interface with different temperature. The negative value of energy indicates that the two layers attract each other. According to the result in Figure 4b, the interaction energy is obviously increased with the increasing temperature. The interaction energy reaches a maximum value of −15542.66 kcal/mol when the temperature increases to 503 K. It is demonstrated that the rise in temperature increases the actual contact area, and further strengthens the interaction between PP droplet and Al substrate. In this present study, no electrostatic interaction between PP and Al atom is formed. All the interaction energy comes from the van der Waals energy. Similar result is also reported that the majority of interaction energy at polyphenylene sulfide/aluminum interface comes from the van der Waals energy, while the electrostatic energy has little contribution [35]. It means the effect of electrostatic energy on the interfacial interaction can be ignored in the interaction energy analysis.

### 3.2. Wetting Behavior of PP Droplet on Rough Surface

**Figure 5:**
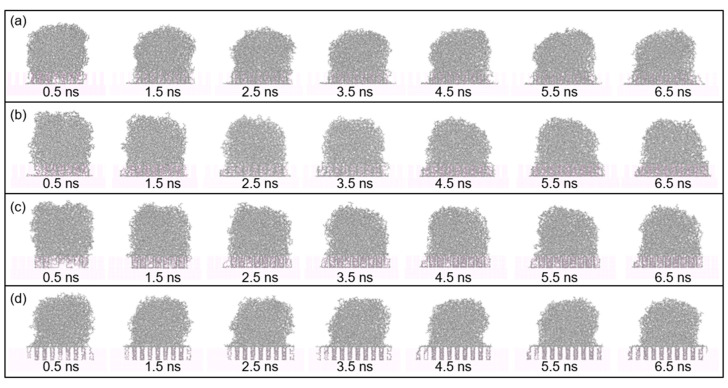
Snapshots of the spreading process of PP droplet with simulation time on Al substrate with rough surface. The side length of the column array was (**a**) 0.40 nm, (**b**) 0.64 nm, (**c**) 0.81 nm and (**d**) 1.21 nm respectively.

In this section, Al substrate with rough surface was constructed to analyze the spreading of PP droplet at the melt temperature of 473 K. Figure 5 demonstrates seven sequential snapshots of the spreading process of PP droplets on different rough surface. It is shown that molecules in PP model are able to penetrate into the gap in the Al substrate, regardless of the side length of column array. Further penetration of molecules in PP model means more contact area for PP-Al interaction, which causes greater interaction energy.

**Figure 6:**
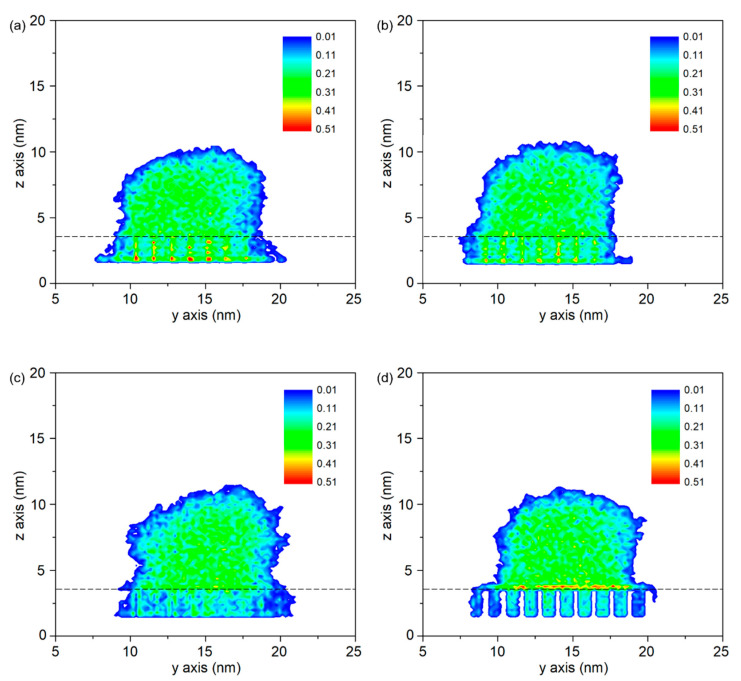
Density distributions of the PP droplets on Al substrate with different rough surfaces. (**a**) column arrays with side length of 0.40 nm (P1), (**b**) column arrays with side length of 0.64 nm (P2), (**c**) column arrays with side length of 0.81 nm (P3) and (**d**) column arrays with side length of 1.21 nm (P4).

Figure 6 shows the density distributions of PP droplets on Al substrate with different rough surfaces. When the side length of the column is 0.40 nm, the corresponding void fraction is 94.6%. The gap between the column arrays is wide enough for PP molecule to penetrate into the substrate. Compared to the other area, high density is observed at the bottom of the column along the height direction (see Figure 6a). It indicates that the molecules in PP model are attached and further enriched on the side surface of nano-column, even though no external force is applied. This is mainly due to the interaction between two layers. In this case, the contact state can be regarded as the Wenzel state. When the side length increases, such phenomenon gradually weakens and eventually disappears. It is calculated that the mean radius of gyration (R_g_) for PP model at initial stage is 1.46 nm, ranging from 0.97 nm to 2.22 nm for a certain chain. By comparing the value of R_g_ and the gap (b) between two columns, it can be found that penetration process become difficult in these cases because the size of these entangled molecule chains is larger than the confined geometry. When the side length of column is 1.21 nm, the gap between two columns is 0.51 nm and the corresponding void fraction reaches to 50.5%, as shown in Table 3. In this situation, only a few molecules in PP model are able to spread along the column and flow into the void with the help of the interfacial interaction force. The contact state is similar to the Cassie–Baxter state at macroscale. As a result, molecules are enriched on the upper surface of the column arrays and a maximum density is observed, as shown in Figure 6d.

In this work, the void fraction and roughness ratio are utilized to define the rough surface, as shown in Table 3. In here, the contact angle is difficult to measure accurately because of the irregular outline of PP droplet. However, by analyzing the density distribution of PP droplet, especially the distribution at the interface gap, it can be found that the contact state is transitioning from Wenzel state to Cassie–Baxter state. The interaction energy also increases with the increase of roughness ratio, from −11,234.2 kcal/mol to −15,009.2 kcal/mol. Although only a few molecules in PP model can be filled into the void in Case P4, the interaction energy reaches to a maximum value due to the increased interaction area.

### 3.3. Dependence of Metal Substrate

**Figure 7:**
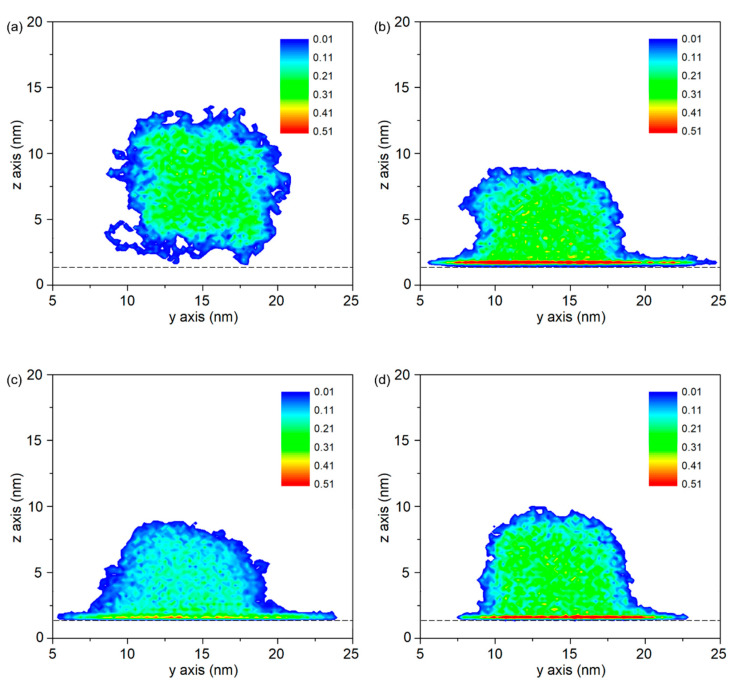
Density distributions of the PP droplet on different substrate. (**a**) initial density of PP droplet on Al substrate, final density distributions of PP droplet on (**b**) Al substrate, (**c**) Ni substrate and (**d**) Fe substrate at the simulation time of 6.5 ns.

Figure 7 presents the density distributions on three different substrates. As shown in Figure 7a, the density distribution is lower than the initial density that is set in the model construction. This is due to the pre-treatment such as geometry optimization, anneal treatments under the non-periodic boundary condition. Without boundary restriction, PP molecules are easy to move outwards, result in an expanded size of PP model. Figure 7b–d show the density distributions at the simulation time of 6.5 ns. It is observed that the maximum densities in PP-Al model, PP-Ni model and PP-Fe model are found at the interface, even higher than the initial density of PP model. It indicates the molecules in PP model are adsorbed on the surface because of the interfacial interaction.

**Figure 8:**
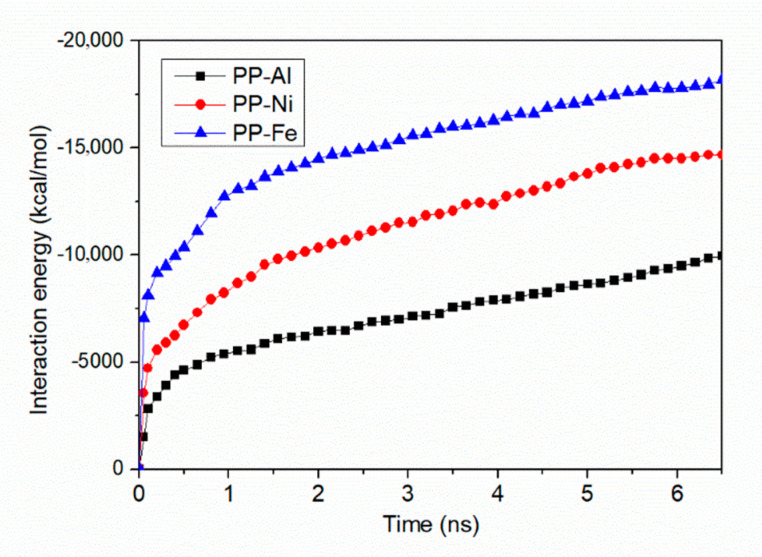
Interaction energies at PP-Al, PP-Ni and PP-Fe interface as a function of simulation time.

Figure 8 shows the interaction energy at the PP-metal interface as a function of simulation time. The contact angles of PP droplet on Ni and Fe substrate are 92.2 ± 3.7° and 124.6 ± 4.5°, which means the PP droplet on the Ni substrate shows the better wetting property. More molecules in PP model are able to generate interaction with the substrate. It is known from other research that no chemical bond between polymer and metal is formed during the joining process, which means the interfacial interaction strength is determined by the non-bonded energy [22]. By comparing the constant value (*ε*) of non-bonded interaction in PCFF force field, the non-bonded interaction between PP and Fe atoms shows highest strength. The constant values (*ε*) are 3.32 kcal/mol, and 5.07 kcal/mol and 13.88 kcal/mol. As a result, the final interaction energies at the interface are −9956.2 kcal/mol for Al-PP system, −14,672.2 kcal/mol for Ni-PP system and −18,162.3 kcal/mol for Fe-PP system, respectively. From perspective of interfacial interaction, the PP-Fe hybrid system has a better potential for the tight joining in direct molding process.

### 3.4. Influence of Metal Substrate on the Joining Strength of Injection-Molded Hybrid Structure

**Figure 9:**
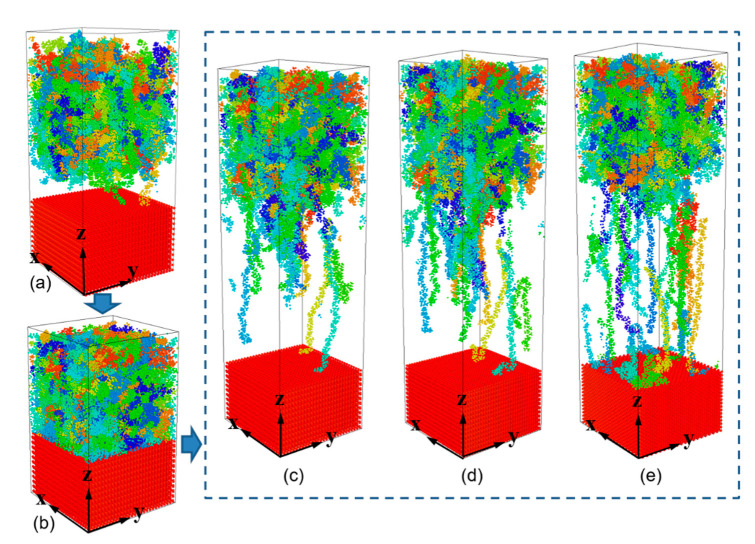
Snapshots of PP-metal hybrid structure during the injection molding and separation process. (**a**) the original PP-metal layer is constructed and then (**b**) injection-molded to form a hybrid system. After cooling process, an external force was applied to remove the PP layer away from the metal substrate. These snapshots are taken at a separation time of 10 ps, with the substrates of (**c**) Al, (**d**) Ni and (**e**) Fe, respectively.

Previous studies indicate that non-bonded interaction plays an important role in the formation of interfacial joining. The combinations of different metal substrates and polymer materials definitely result in different joining strengths. In order to further explore the influence of interfacial interaction on the joining property of polymer-metal hybrid structure, the direct joining process was undertaken. As shown in Figure 9a, the same PP layer mentioned in the Section 2.1, was used for the construction of PP-metal hybrid structure. After that, the PP melt was injected into the mold cavity by applying an injection force of 0.16 kcal/mol∙A along the height direction (see Figure 9b). After the cooling process, an external force of 0.1 kcal/mol∙A was applied in order to remove the PP layer away from the metal substrate. The metal substrate was treated as the rigid body during the separation. More details on the simulation procedure can be found in the previous research [29].

Figure 9c–e displays the snapshots of PP-metal hybrid system at a separation time of 10 ps. The substrates are Al, Ni and Fe, respectively. It is clearly shown that molecule chains near the interface are greatly stretched along the height direction, the length of certain chains can be more than ten nanometers. While molecule chains far away from the interface show little conformation changes. For both PP-Al and PP-Ni hybrid structures, we can find that the PP layers are totally separated from the substrates at the end of stretching process. It is demonstrated that interfacial failure occurs in terms of PP-Al and PP-Ni system. For PP-Fe hybrid structure, more simulation time is required for the separation and more molecular chains are stretched, as shown in Figure 9e. The majority of the PP chains are pulled out from the Fe substrate, while some still remain close to the surface, which means cohesive failure occurs in the polymer bulk.

**Figure 10:**
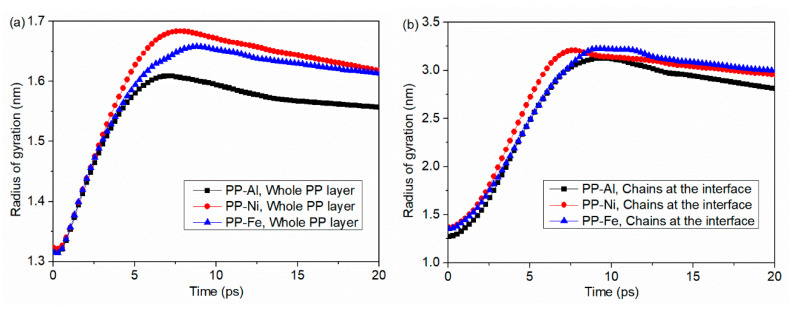
Radii of gyration in PP layer during the separation process with different substrate structures. The radius of gyration is calculated for (**a**) the whole PP layer and (**b**) molecular chains at the interface that are obviously stretched during the separation.

**Figure 11:**
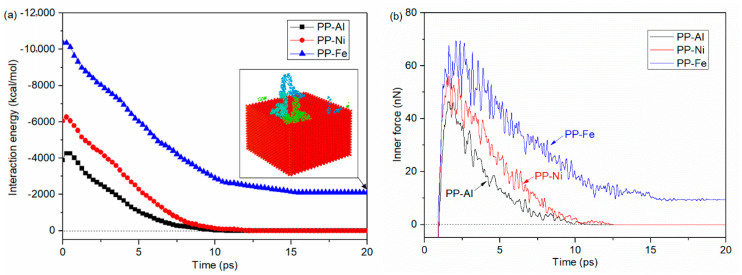
(**a**) interaction energies of injection-molded polymer-metal hybrid structure and (**b**) inner forces in PP layer during the separation process with different substrates.

Radius of gyration in PP layer during the separation process was calculated, as shown in Figure 10. It is demonstrated in Figure 9 that the chains are stretched away from the substrate until the polymer layer is removed away from the substrate. As a result, the radius of gyration first increases rapidly and afterwards decreases due to the elastic recovery of stretched chains. Therefore, a distinct peak is observed before the complete separation moment. Moreover, the change in radius of gyration mainly comes from the conformation change near the interface, as shown in Figure 10b. In the case of PP-Fe system, the gyration radius for the whole PP layer increases from 1.32 nm to 1.66 nm, increased by 25.76%. Meanwhile, the gyration radius of chains at the interface increases from 1.35 nm to 3.23 nm, increased by 139.26%. A higher conformation change in the radius of gyration means that the chains are more stretched because of the combination effects of external pull-out force and non-bonded interaction at the interface. As seen in Figure 9e and Figure 11a, some molecule chains remain close to the Fe surface finally, leading to the result that the change in radius of gyration in PP-Fe system is relatively lower than that in PP-Ni system, as shown in Figure 10a.

The inner force-time curves indicate the inner behaviors during the separation process that is used in the previous reports to analyze the interfacial joining properties [36,37]. Figure 11 shows the interaction energies of polymer-metal hybrid structure and inner forces in PP layer during the separation process with different substrates. By comparing the generated interaction energy with Figure 8, the interaction energies per unit area of the injection-molded hybrid system obviously increase, which are 60.74 kcal/mol∙nm^2^ for PP-Al hybrid structure, 94.54 kcal/mol∙nm^2^ for PP-Ni hybrid structure and 161.74 kcal/mol∙nm^2^ for PP-Fe hybrid structure. The interaction energy per unit area in the wetting simulation model are 24.89 kcal/mol∙nm^2^ for PP-Al interface, 36.68 kcal/mol∙nm^2^ for PP-Ni interface and 45.40 kcal/mol∙nm^2^ for PP-Fe interface. This is because the injection force is applied to the PP layer and molecules are pushed towards the interface, which means more interactions are generated, finally. With the molecules in PP layer removing far away from the substrate, no interfacial interaction will be formed, and the interaction energy turns out to be zero. The only exception is the situation in PP-Fe system, since there are still some molecules adhered on the surface, as shown in Figure 10a. it is found in Figure 11b that inner force first increases dramatically, reaching the highest value within 2.5 ps. With further removal of injection-molded PP layer, the inner force gradually reduces, indicating fewer atoms in polymer layer are available to interact with Al atoms. After an oscillating downward phase, the inner force drops to zero or a relatively steady value, corresponding to the end of separation for the initial layer. By comparing the results with different substrates, the inner force of PP-Fe hybrid structure during the separation process is obviously higher. More time is required to realize a complete separation. These results are consistent with the wetting property and the interaction energy analysis at PP-Al interface as mentioned in the Section 3.3.

## 4. Conclusions

In this paper, MD simulations for wetting process of PP droplet on metallic substrate were performed to study the effects of melt temperature, surface roughness and substrate material on the wetting property and the interfacial interaction energy. Lately, the separation process for the injection-molded PP-metal hybrid structure was then simulated. The joining strength induced by different substrate materials were analyzed. The main conclusions of this work are as follows:

(1) Molecules in PP model are easier to spread along the surface with a higher temperature. At the beginning, the PP droplet forms a sphere-like structure while the atoms in the bottom of PP model start to spread out along the surface. With the time goes on, the whole model gradually collapses and more atoms are attached to the Al surface. The decrease in the height of mass center reflects an increase in the spreading ability of PP droplet.
(2) Molecule chains are able to penetrate into the gap in the rough substrate. High density is observed at the bottom of the column or on the upper surface of the column arrays. The contact state is transitioning from Wenzel state to Cassie–Baxter state with the decrease of void fraction. The interaction energy increases with the increased interaction area.
(3) Maximum densities in PP-Al model, PP-Ni model and PP-Fe model are found at the interface, indicating the molecules are adsorbed on the surface. From perspective of interfacial interaction, the PP-Fe hybrid system have a higher potential for the tight joining in direct molding process.
(4) During the separation process, molecule chains near the interface are greatly stretched, with the highly increased radius of gyration. While molecule chains far away from the interface show little conformation changes. For both PP-Al and PP-Ni hybrid structures, the PP layers are totally separated from the substrates finally. For PP-Fe hybrid structure, the majority of the chains are pulled out from the substrate, while some still remain close to the surface. The inner force of PP-Fe hybrid structure during the separation is obviously higher than other cases.

In the future work of PP-metal interfacial interaction, we will consider the semicrystalline structure of PP that is closer to industrially used materials. Besides, other MD methods such as coarse grained (CG) MD method will be considered to achieve an increase of orders of magnitude in the simulated time and length scales.

## Figures and Tables

**Table 1:** Main parameters of polypropylene (PP) atomistic model.

Material	Degree of Polymerization	Number of Chains	Total Amount of Atoms	Initial Density (g/cm^3^)	Model Size (nm)
PP	50	120	54,240	0.85	8.0 × 8.0 × 8.0

**Table 2:** Main parameters of metal substrates with smooth surface and rough structure. The total amount of atom is counted according to the model from the Al substrate.

Substrate Structure	Side Length (nm)	Height (nm)	Distance (nm)	Total Amount of Atoms	Model Size (nm)
Case P0	/	/	/	39,764	20.0 × 20.0 × 1.4
Case P1	0.40	2.02	1.72	48,532	20.0 × 20.0 × 3.4
Case P2	0.64			53,572	
Case P3	0.81			60,052	
Case P4	1.21			76,012	

**Table 3:** Side length, void fraction, roughness ratio and interaction energy at 6.5 ns with different substrate structures on Al surface.

Substrate Structure	Side Length (nm)	Void Fraction	Roughness Ratio	Interaction Energy (kcal/mol)
Case P1	0.40	94.6%	2.09	–11,234.2
Case P2	0.64	86.2%	2.75	–12,668.1
Case P3	0.81	77.8%	3.21	–12,702.0
Case P4	1.21	50.5%	4.30	–15,009.2
